# Novel Human Reovirus Isolated from Children with Acute Necrotizing Encephalopathy

**DOI:** 10.3201/eid1708.101528

**Published:** 2011-08

**Authors:** Louise A. Ouattara, Francis Barin, Marie Anne Barthez, Bertrand Bonnaud, Philippe Roingeard, Alain Goudeau, Pierre Castelnau, Guy Vernet, Gláucia Paranhos-Baccalà, Florence Komurian-Pradel

**Affiliations:** Author affiliations: Fondation Mérieux, Lyon, France (L.A. Ouattara, G. Vernet, G. Paranhos-Baccalà, F. Komurian-Pradel); Centre Hospitalier Universitaire Bretonneau (F. Barin, P. Roingeard, A. Goudeau);; Institut National de la Santé et de la Recherche Médicale U966, Tours, France (F. Barin, P. Roingeard, A. Goudeau);; Centre Hospitalier Universitaire, Clocheville, Tours (M.A. Barthez, P. Castelnau);; bioMérieux SA, Marcy l’Etoile, France (B. Bonnaud)

**Keywords:** encephalitis, mammalian orthoreovirus 2, reassortant viruses, viruses, research

## Abstract

For many encephalitis cases, the cause remains unidentified. After 2 children (from the same family) received a diagnosis of acute necrotizing encephalopathy at Centre Hospitalier Universitaire (Tours, France), we attempted to identify the etiologic agent. Because clinical samples from the 2 patients were negative for all pathogens tested, urine and throat swab specimens were added to epithelial cells, and virus isolates detected were characterized by molecular analysis and electron microscopy. We identified a novel reovirus strain (serotype 2), MRV2Tou05, which seems to be closely related to porcine and human strains. A specific antibody response directed against this new reovirus strain was observed in convalescent-phase serum specimens from the patients, whereas no response was observed in 38 serum specimens from 38 healthy adults. This novel reovirus is a new etiologic agent of encephalitis.

## Abstract 2

TOC summary line: This new virus may be another cause of encephalitis.

Mammalian reoviruses, members of the genus *Orthoreovirus*, are nonenveloped double-stranded RNA viruses with a genome composed of 10 segments. These viruses have 3 major serotypes: type 1 Lang (T1L), type 2 Jones (T2J), and type 3 Dearing (T3D), which can be differentiated by neutralization and hemagglutination inhibition tests (*1*). A fundamental characteristic of these viruses, because of their segmented genome, is that 2 distinct viruses can infect the same cell and combine their genomes, thus generating novel viruses (*2*). The acronym reovirus (respiratory enteric orphan virus) is used to designate viruses isolated from the respiratory and enteric tracts of persons with mild respiratory or gastrointestinal symptoms (*3*). Although reovirus infection of humans usually induces mild symptoms, infection of newborn mice leads to severe pathologic conditions, such as lethal encephalitis, depending on the inoculation route and strain (*4**,**5*). Previous studies have described the isolation of 3 reovirus strains after cell culture of cerebrospinal fluid (CSF) from patients with meningitis: serotype 1 (*6*), serotype 3 (T3C96) (*7*), and serotype 2 (T2W) (*8**,**9*). The etiologic role of the T2W strain in meningitis could not be ascertained because the patient was co-infected with other agents (*8*). Similarly, new mammalian reoviruses, such as BYD1, JP, and BYL, were isolated from throat swab specimens of patients with severe acute respiratory syndrome (*10*). Similarly, Melaka virus (*11*), Kampar virus (*12*), and HK23629/07 virus (*13*) were isolated from adults with acute respiratory infection. Here, we report the isolation of a novel human type 2 reovirus (named MRV2Tou05) from 2 children hospitalized with acute necrotizing encephalopathy (ANE). Virologic, molecular, and serologic methods were used to detect the MRV2Tou05 strain.

## Patients and Methods

### Patients

A 6-year-old boy (patient 1) and his 22-month-old cousin (patient 2) were hospitalized with the same ANE-specific symptoms a few days apart in March–April 2005 in Centre Hospitalier Universitaire, Clocheville (Tours, France). Serum, urine, CSF, and throat swab specimens were collected from both children. An influenza-like syndrome developed simultaneously in the mother of patient 2.

### Laboratory Procedures

To detect herpesviruses and enteroviruses, we used PCR or reverse transcription PCR (RT-PCR), respectively, with commercially available reagents (herpes consensus generic detection kit and enterovirus consensus detection kit [Argene, Verniolle, France]). Nasopharyngeal aspirates were tested by indirect immunofluorescence, by using specific monoclonal antibodies (Argene), for influenza viruses A and B, respiratory syncytial virus, parainfluenza viruses 1–3, and adenoviruses. Serologic assays (ELISA) with commercially available reagents (Behring, Paris, France) were conducted to detect immunoglobulin (Ig) G and IgM against herpes simplex viruses, Epstein-Barr virus, measles virus, and mumps virus. Antibodies to influenza viruses were tested by complement fixation assays with antigens derived from influenza viruses A and B. Serologic assays were also conducted to detect IgG and IgM against hantavirus, tick-borne encephalitis virus, dengue virus, and chikungunya virus (National Reference Center for Arboviruses, Institut Pasteur, Paris). In addition, because the 2 patients had been in contact with an uncle who had returned from Asia (Indonesia), the presence of Hendra virus and Nipah virus nucleic acid sequences in CSF was investigated.

### Virus Isolation, Propagation, and Identification

Urine and throat swab specimens from each patient were added to MRC5, MDCK, and Vero cells. Early viral stocks were made from urine and throat specimens added to MRC5 cells as soon as cytopathic effects (CPEs) were observed. Late viral stocks were obtained after inoculation of BGM cells. Cells were harvested as soon as CPEs were observed and fixed by incubation for 48 h in 4% paraformaldehyde and 1% glutaraldehyde in 0.1 mol/L phosphate buffer, pH 7.2, as described (*14*). Cell pellets were embedded in Epon resin (Sigma-Aldrich, Saint-Quentin Fallavier, France), which was allowed to polymerize for 48 h at 60°C. Ultrathin sections were cut, stained with 5% uranyl acetate and 5% lead citrate, and deposited on electron microscopy grids that were coated with collodion membrane for examination under a Jeol 1010 transmission electron microscope.

### Genome Virus Amplification and Sequencing

Specific primers of each reovirus segment were constructed in the highly conserved regions (Table 1). The PCR products corresponding to large (L), medium (M), and small (S) segments were cloned and sequenced in both directions. Sequence analysis was conducted by using Vector NTI (Invitrogen, Carlsbad, CA, USA). To obtain the S1 segment, we extracted total RNA from infected BGM cells with TRIZOL reagent (Invitrogen). The double-stranded RNA was obtained as previously described (*15*) and separated through migration on a 6% precast Poly(NAT) gel (Elchrom Scientific AG, Cham, Switzerland). A ≈1,400-bp band was recovered from the gel and directly used for RT-PCR with random primers (Invitrogen and Roche [La Rochelle, France]). The PCR products were purified from the gel and cloned, and several clones were sequenced. To complete the S1 sequencing, a specific S1 primer was constructed and used for the RT step. The amplification was done by using the specific S1 primer and a random primer (Roche). The band corresponding to the expected size (684 bp) was purified from the gel, cloned, and sequenced. The new sequences enabled construction of specific primers at each extremity (Table 1) to amplify the entire S1 segment and sequence in both directions.

**Table 1:** Primers used for amplification of MRV2TOU05, a novel human type 2 reovirus, and complete sequencing and reovirus detection*

Primer	Sequence, 5′ → 3′	Position (strain)	RT-PCR product size, bp	GenBank accession no.
For genome amplification of MRV2Tou05				
L1 forward	GCTACACGTTCCACGACAAT	1–20 (SC-A)	3,852	GU196306
L1 reverse	TGAGTTGACGCACCACGACCCA	3852–3831 (SC-A)		
L2 forward	ATGGCGAACGTTTGGGGAGT	13–32 (SC-A)	3,903	GU196307
L2 reverse	GATGAATTAGGCACGCTCACG	3915–3895 (SC-A)		
L3 forward	TAATCGTCAGGATGAAGCGGA	3–23 (SC-A)	3,897	GU196308
L3 reverse	TGAATCGGCCCAACTAGCAT	3899–3880 (SC-A)		
M1 forward	ATGGCTTACATCGCAGTTCCT	14–34 (SC-A)	2,278	GU196309
M1 reverse	CGTAGTCTTAGCCCGCCCC	2291–2273 (SC-A)		
M2 forward	TAATCTGCTGACCGTCACTC	3–22 (SC-A)	2,195	GU196310
M2 reverse	GTGCCTGCATCCCTTAACC	2197–2179 (SC-A)		
M3 forward	CGTGGTCATGGCTTCATTC	12–30 (SC-A)	2,230	GU196311
M3 reverse	GATGAATAGGGGTCGGGAA	2241–2223 (SC-A)		
S2 forward	CTATTCGCTGGTCAGTTATG	2–21 (SC-A)	1,330	GU196312
S2 reverse	GATGAATGTGTGGTCAGTCG	1331–1312 (SC-A)		
S3 forward	TAAAGTCACGCCTGTTGTCG	3–22 (SC-A)	1,178	GU196313
S3 reverse	ACCACCAAGACATCGGCAC	1180–1162 (SC-A)		
S4 forward	GTTGTCGCAATGGAGGTGTG	24–43 (SC-A)	1,158	GU196314
S4 reverse	TCCCACGTCACACCAGGTT	1181–1163 (SC-A)		
S1 forward	CCGATGTCCGAACTTCAACA	1–17 (MRV2Tou05)	1,423	GU196315
S1 reverse	ATGAATTGCCGTCGTGCCG	1423–1405 (MRV2Tou05)		
For reovirus detection test				
L3-2 reverse	GGATGATTCTGCCATGAGCT	705–686 (BYD1)	696	ND
L3-1 forward	CAGGATGAAGCGGATTCCAA	10–29 (T3D, T1L, T2J, SC-A, BYD1)		ND
L3-5 reverse	CCAACACGCGCAGGATGTTT	522–503 (T3D, BYD1, T1L)	512	ND
L3-1 forward	CAGGATGAAGCGGATTCCAA	10–29 (T3D, T1L, T2J, SC-A, BYD1)		ND

*RT-PCR, reverse transcription PCR; L, large segment; M, medium segment; S, small segment; ND, not determined.

### Phylogenetic Analysis

Sequence alignments (nucleic acid and amino acid) were constructed using ClustalW 1.74 (*16*) and refined by visual inspection with SEAVIEW; distance matrix and phylogenetic trees were computed with PHYLO_WIN (*17*). Distances between sequences were computed by observed divergence. Trees were built by using the neighbor-joining method, and tree topologies were tested with 1,000 bootstrap sampling replicates.

### Antireovirus IgG Detection

BGM cells infected with the MRV2Tou05 strain and noninfected cells were used for Western blot analysis with a 1:100 dilution of the 2 patients’ serum specimens. Thirty-eight serum specimens (supplied by the Établissement Français du Sang, Lyon, France) obtained from 38 healthy blood donors were tested by Western blot for antibodies against the MRV2Tou05, Lang (T1L), Jones (T2J), and Dearing (T3D) strains.

### Reovirus RNA Detection

Viral RNA was extracted from infected cells and supernatant and from patients’ samples by using the Nuclisens EasyMAG Kit (bioMérieux). Mock cells and supernatant from uninfected cells were used for RNA extraction as negative controls. A molecular test was set up with the L3 segment as target with primers enabling detection of the 3 reovirus serotypes (Table 1).

## Results

### Patient Histories

**Figure 1:**
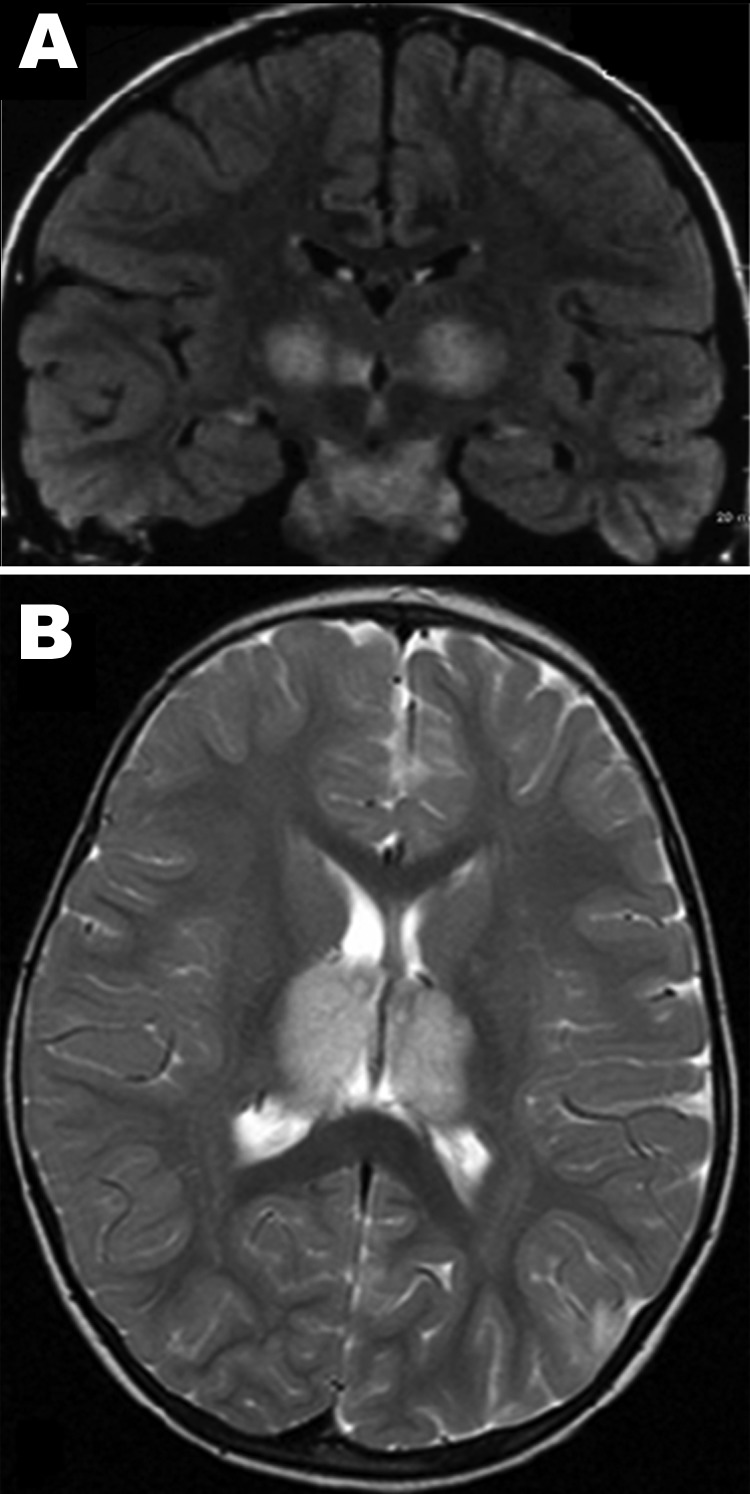
A) Magnetic resonance image of brain corona of patient 1, a 6-year-old boy with acute necrotizing encephalopathy (ANE). B). Axial-weighted images of brain thalami of patient 2, a 22-month-old girl with ANE, the cousin of patient 1.

Patient 1, a previously healthy 6-year-old boy, was admitted to Centre Hospitalier Universitaire (Tours) on March 29, 2005, with fever, vomiting, and impaired consciousness. Results of his neurologic examination were normal, except for palsy of the left facial nerve. The Glasgow Coma Scale score was 7. He could respond to simple verbal commands, such as requests to open eyes or make some other movements, but he was lethargic (no articulated language) and had epilepsy-like abnormal movements of the face or distal muscles. For 2 days previously, he had experienced a prodromal illness with high temperature (40°) and headache. Results of an analysis of CSF on the first day of hospitalization were within normal limits and showed a protein level of 0.81 g/L and 4 leukocytes/mm^3^ on day 5. An electroencephalogram showed generalized slow-wave activity. Computed tomography of the brain showed a low-density change in the thalami. Brain magnetic resonance imaging (MRI) performed on the first day of hospitalization showed multiple symmetric lesions, with high signal intensity on T2-weighted images and low signal intensity on T1-weighted images, mostly involving the thalami bilaterally but also the brainstem tegmentum (Figure 1, panel A) and cerebral white matter in external capsules. He received treatment in the intensive care unit and was given acyclovir, methylprednisolone, and clonazepam intravenously. Beginning 2 days after he arrived at the hospital, he showed improvement, and he recovered completely in a few weeks. He was discharged on the 25th day of illness. A follow-up brain MRI, 1 year later, showed no abnormal findings. Three years later, his development and neurologic examination results were within normal limits.

Patient 2, a 22-month-old girl, was the cousin of patient 1. She was admitted 4 days later, on April 3, 2005, with drowsiness and fever after 1 day of high fever, asthenia, and rhinorrhea. A neurologic examination showed brisk deep tendon reflexes and extensor plantar responses; Glasgow Coma Score was 8. CSF initially showed a slightly elevated protein level (0.55 g/L, reference <0.30 g/L) with no cells, and findings were within normal limits on day 4 of illness (0.38 g/L protein, no cells). Radiologic examinations of the brain showed similar lesions to those of her cousin (Figure 1, panel B), and she received the same treatment. She experienced mild hepatomegaly on day 11 of illness. Her condition began to improve on day 7 of illness, and she was discharged from the hospital on day 18, April 20, with marked hypotonia, responsiveness but absence of language, and pyramidal tract signs. One year later, she remained easily tired, with uncoordinated movements. MRI of the brain found that the damage was less visible and the signal was much less marked.

Two other family members (the mother of patient 2 and the grandmother of both children) also had influenza-like symptoms (headache, fever, vomiting) at the same period for a few days without neurologic signs. The past family history and medical history did not appear relevant.

### Initial Virologic Investigation

Results of molecular tests for herpesviruses and enteroviruses performed initially on the CSF specimens from both children were negative. The results were also negative for infection of respiratory viruses (influenza viruses A and B, respiratory syncytial virus, parainfluenza viruses 1–3, and adenoviruses). Serologic test results for HIV were negative. Serologic assays for IgG and IgM against herpes simplex virus, Epstein-Barr virus, measles virus, and mumps virus did not show IgM. Serologic assays were negative for both IgG and IgM against hantavirus, tick-borne encephalitis virus, dengue virus, and chikungunya virus. The results were also negative for nucleic acid sequences of Hendra virus and Nipah virus (F. Wild, National Reference Center for Measles and Other Paramyxoviruses, pers. comm.). All serum specimens collected during the acute and convalescent phases were negative for antibodies against influenza viruses.

### Virus Isolation and Propagation

**Figure 2:**
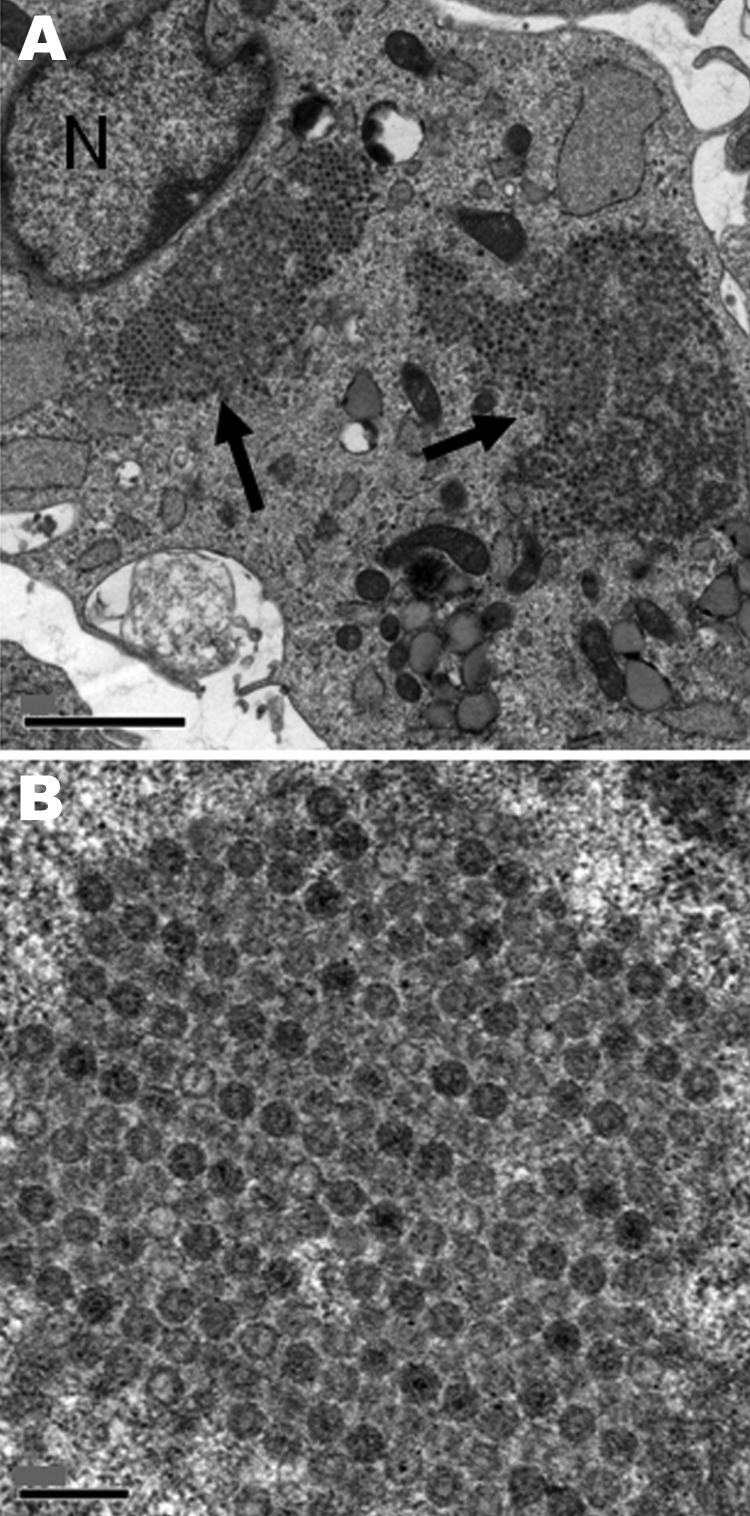
Electron microscopic images of the cytopathic effect induced in MRC5 cells by a reovirus isolate from throat specimens of patient 2, a 22-month-old girl with acute necrotizing ancephalopathy. N, nucleus; arrows indicate viral intracytoplasmic inclusions. Scale bars indicate 2 µm (A) or 0.2 µm (B).

Nonoriented virus isolation was attempted by inoculation of MRC5, MCDK, BGM, and Vero cell cultures with urine collected from each patient on April 7 and with a throat swab specimen from patient 2 on April 11. A CPE was observed on day 7 in MRC5 cells inoculated with urine from both patients, and on day 7 in MCR5 cells and day 10 in MDCK cells inoculated with a throat swab specimen from patient 2. A discrepant result for enterovirus was obtained when identification was attempted. Indeed, indirect immunofluorescence on fixed cells was positive for enterovirus (Pan-Enterovirus Blend, Light Diagnostics [Millipore, Molsheim, France]), but results of RT-PCR for detection of enteroviruses, performed on the cell culture supernatant, were negative. The virus isolates were then identified as reovirus type 2 by seroneutralization at the National Reference Center for Enteroviruses (Lyon, France). The discrepancy initially observed between immunofluoresence and RT-PCR results may be attributed to the cross-reactivity of the Pan-Entero Blend reagent toward reoviruses, as mentioned in the manufacturer’s description. Microscopic examination after staining of infected cells showed voluminous cytoplasmic inclusions characteristic of CPEs induced by reoviruses; electron microscopy showed the accumulation of virions in formation (Figure 2).

### Reovirus Detection in Patients’ Specimens

Reovirus sequences were searched for retrospectively by RT-PCR in the patients’ available specimens. A specific 512-bp fragment corresponding to the L3 expected region was obtained from urine specimens from both patients and from 1 serum specimen that was obtained 21 days after the onset of symptoms from patient 1 (data not shown). All amplified fragments were sequenced and showed identical profiles.

### Molecular and Phylogenetic Characterization

The complete sequence of the MRV2Tou05 genome was determined in both directions. Nucleotide and deduced amino acid sequences obtained for each segment were analyzed in the National Center for Biotechnology Information database (www.ncbi.nlm.nih.gov/Database/) to determine the percentage of identity with mammalian reoviruses. Sequence analysis of the 3 large segments, the 3 medium segments, and the S2, S3, and S4 segments indicated overall a close genetic relationship between MRV2Tou05 and swine SC-A viruses and a relationship with the 2 reovirus prototypes 1 and 3 (Table 2). The sequences of the MRV2Tou05 S1 gene showed great diversity at the nucleotide and amino acid levels, compared with the S1 gene from mammalian reoviruses and SC-A strains (Table 2, Table 3). Otherwise, the S3 gene showed high identity with type 2 human reovirus strains (Table 3). The highest identities (83% and 99%) were with type-2 human isolates 302I and 302II from the People’s Republic of China (*18*) and human isolates from the Netherlands (*18*), respectively, for S1 and S3 genes (Table 3). Nucleotide sequences of the prototype T2J and the novel MRV2Tou05 S1 genes shared 62% of positional identity, which provided sequence confirmation that this new isolate was a type 2 strain.

**Table 2:** Nucleotide and amino acid identities for segments of MRV2Tou05, a novel human type 2 reovirus, isolated from patient 2, with reovirus prototypes and SC-A strain*

MRV2Tou05 RNA segment	Reovirus prototype strain, %								Swine reovirus strain, SC-A, %	
	T1L			T2J			T3D			
	Nucleotide	Protein		Nucleotide	Protein		Nucleotide	Protein	Nucleotide	Protein
L1	90	98		75	92		90	98	97	99
L2	86	97		73	87		77	93	97	98
L3	84	98		77	95		84	98	97	99
M1	92	97		70	80		92	96	90	95
M2	85	98		76	97		90	98	97	99
M3	85	95		71	82		85	95	97	98
S1	58	46		62	62		42	26	42	25
S2	85	98		76	94		85	98	88	98
S3	91	97		74	86		85	97	91	99
S4	87	97		79	91		87	96	95	99

*T1L, type 1 Lang; TJ2, type 2 Jones ; T3D, type 3 Dearing; L, large segment; M, medium segment; S, small segment.

**Table 3:** Comparison of S1 and S3 genes of MRV2Tou05, a novel human type 2 reovirus, with the most representative mammalian reovirus strains*

Strain	GenBank accession no.			% Similarity to MRV2Tou05 S1			% Similarity to MRV2Tou05 S3	
	S1	S3		Nucleotide	Protein		Nucleotide	Protein
T1C50	AY862133	NA		57	50		NA	NA
T1N84	AY862136	NA		58	53		NA	NA
T1N85	AY862135	U35346		57	51		98	99
T1C11	NA	U35359		NA	NA		91	98
T1C62	NA	U35356		NA	NA		91	99
T1C23	AY862134	NA		57	50		NA	NA
T2N73	AY862137	U35350		67	66		99	99
T2N84	AY862138	U35347		67	66		98	99
T2W	DQ220017	DQ220018		66	62		75	88
T2302II	EU049604	NA		83	89		NA	NA
T2302I	EU049603	NA		83	89		NA	NA
BYD1	DQ312301	DQ664191		66	67		84	95
SC-A	DQ911244	DQ411553		42	25		91	97
T3Co96	AY302467	NA		43	28		NA	NA
T3A	L37677	NA		48	25		98	97
T3C18	L37684	NA		42	26		NA	NA
T3C8	L37679	U35355		42	24		85	97
T3C31	L37683	NA		41	25		NA	NA
T3C9	L37676	U35352		40	25		82	97
T3C93	L37675	NA		42	25		NA	NA
T3C44	L37681	NA		42	26		NA	NA
T3C45	L37680	NA		42	25		NA	NA
T3C43	L37682	NA		42	25		NA	NA
T3C84	L37678	U35354		42	25		85	97
T3N83	NA	U35349		NA	NA		85	98

*S, small segment; NA, not available.

**Figure 3:**
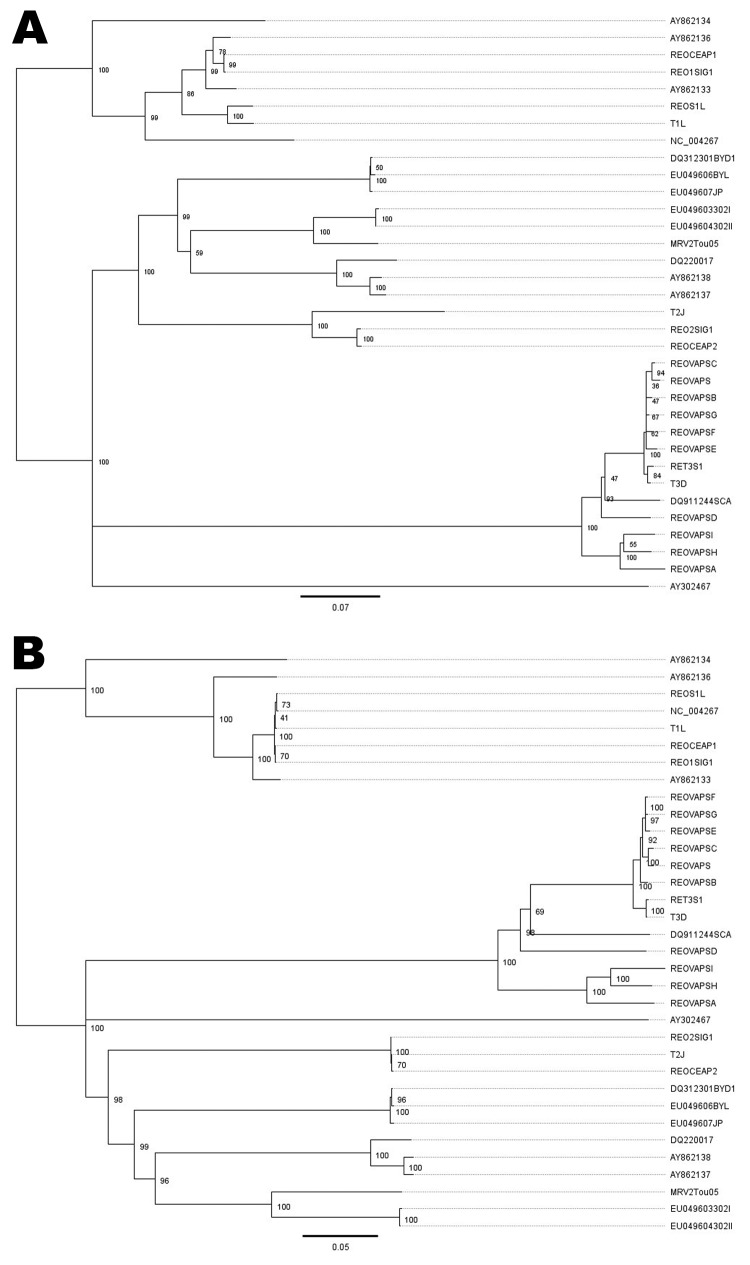
Phylogenetic trees of the small segment 1 of reoviruses. A) Nucleotide sequences; B) amino acid sequences. Scale bars indicate nucleotide (A) and amino acid (B) substitutions per site.

To establish the evolutionary relationship of MRV2Tou05 with the known mammalian reoviruses, we constructed phylogenetic trees on the basis of the nucleotide sequences of the S1 and S3 segments (Figure 3). The S1 phylogenetic tree shows that the MRV2Tou05 S1 sequence is more closely related to the S1 genes of other type 2 reovirus strains and most divergent from the type 3 reovirus strains, including the SC-A strain. In contrast to the great diversity of the MRV2Tou05 S1 gene sequence compared with the other reovirus strains, similar trees obtained with sequences of the MRV2Tou05 S2, S3, and S4 genes showed a randomized genetic clustering between the different reovirus types for these genes (data not shown).

The 4 S segments, S1–S4, of MRV2Tou05, isolated from the throat swab specimen of patient 2 and the urine specimen of patient 1, were entirely sequenced. The nucleotide sequence was identical for 62 of 81 clones, and <3 point mutations were observed among the whole RNA segments for the remaining 19 clones, indicating that the 2 children had been exposed to the same novel isolate.

### Serologic Analysis

**Figure 4:**
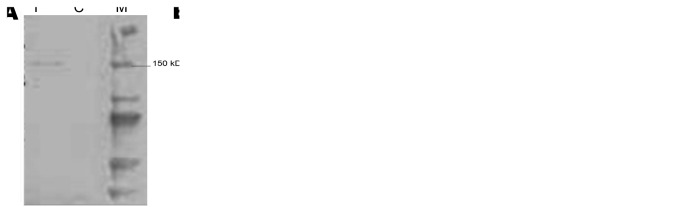
Results of serologic analysis by Western blot of serum specimens from patient 2, a 22-month-old girl with acute necrotizing ancephalopathy. Three serum specimens from patient 2, harvested at 6 (A), 13 (B), and 19 (C) days after onset of symptoms, and a serum specimen from a healthy donor (D) were incubated with reovirus MRV2Tou05–infected and –noninfected BGM cells. I, infected; C, noninfected; M, molecular weight markers (Precision Plus protein standards)**.**

Specific antibodies against MRV2Tou05 were detected in serum specimens from patient 2; reactivity was higher in the 2 specimens collected in the convalescent phase, i.e., 13 and 19 days after symptom onset (Figure 4, panels B, C) than in specimens collected in the acute phase (6 days after symptom onset) (Figure 4, panel A). Similarly, antibodies against MRV2Tou05 were detected in the serum specimens from patient 1 and the mother of patient 2 (data not shown). The molecular mass of 145 kDa corresponded to λ proteins encoded by the large segments, with a size of 142–145 kDa. None of the 38 serum specimens from healthy blood donors was positive for MRV2Tou05 reovirus (Figure 4, panel D) but 20 of 38 serum specimens from donors were positive for the reovirus prototype strains, with the highest prevalence for T1L and T3D (data not shown). In the donors’ blood, we observed a signal directed against the λ proteins as well as 2 additional bands corresponding to the μ proteins (75 kDa and 100 kDa) from the human reovirus prototypes. Antibodies against T1L and T3D strains were also found in serum specimens from patient 2, but not in specimens from patient 1 and from the mother of patient 2 (data not shown).

## Discussion

For many cases of encephalitis (32%–75%), the etiologic agent remains unknown (*19**–**21*). In this study, a reovirus strain was isolated from 2 children who exhibited unexplained neurologic symptoms of encephalitis. On the basis of the neuroimaging findings, clinical features, and laboratory data, ANE was diagnosed (*22*). The viruses commonly involved in encephalitic syndromes were not detected in CSF specimens from both patients. Results of broad virologic investigations for other viruses were also negative. The reovirus isolation and the molecular detection of the specific sequences in patients’ specimens demonstrated that the 2 hospitalized children with ANE had been exposed to a novel reovirus strain. Tyler et al. (*7*) recently described a novel type 3 reovirus isolated from a child with meningitis and provided direct evidence that reoviruses can be neurovirulent in humans.

Sequence analysis and phylogenetic trees showed that most reovirus segments (L1–L3, M2–M3, S2, and S4) were closely related to the swine reovirus strain (91%–97% identity), except for the S1 and S3 segments. The S1 segment determines the reovirus serotype and encodes the nonstructural protein sigma 1s and the viral cell attachment protein sigma 1. The S1 gene showed a high identity score (83%) with human type 2 strains isolated from fecal specimens of 2 children in China in 1982 (*18**,**23*). This finding was compatible with the results of the neutralization assay performed with the initial infected cell culture supernatant. The S1 segment from reoviruses is highly diverse in size and sequence. This diversity may explain why, even within the same serotype, the S1 segment could not be amplified with a different set of primers initially defined by alignment of reovirus serotype 2 strains. The S3 segment is genetically similar to that of the human reovirus strains T2Neth/73 and T2Neth/84 (98% identity) isolated in the Netherlands in 1973 and 1984 (*24*). The S3 gene is known to encode the nonstructural protein σNS (*25**,**26*), which plays a notable role in the replication of reovirus gene segments and assortment (*27**,**28*). On the basis of these data, we conclude that the novel type 2 human reovirus, designated MRV2Tou05, might have originated from a reassortment between a human isolate and a swine reovirus, both probably first identified in China.

A specific antibody response against the MRV2Tou05 strain developed in the 2 patients and in the mother of patient 2, whereas no antibody response against MRV2Tou05 was detected in any of the 38 healthy blood donors. However, serum samples from 52% of these healthy adults contained antibodies directed against at least 1 of the 3 human reovirus prototype strains. In a study in Germany, similar seroprevalence was observed for reovirus type 3 antibodies in a healthy population (*29*). Although the studied population is limited, our data suggest that isolates related to the reovirus prototype strains are spread widely in the human population, in contrast to this novel type 2 MRV2Tou05 reovirus.

ANE predominantly affects infants and young children in eastern Asia, but sporadic cases are regularly diagnosed in other parts of the world. The most frequent pathogens involved in ANE are viruses, most commonly influenza A and B (*22**,**30*). The outcome of ANE has been reported to be generally poor, but the 2 patients described in this study recovered and had only mild sequelae or none (*31**,**32*). Influenza-associated encephalopathy has been mostly reported in children in Japan and Taiwan, which suggests a possible association with genetic or epigenetic factors in these countries (*30*). A similar disorder has been described as autosomal dominant ANE with incomplete penetrance in only 1 large family. It affects children, and clinical and radiologic findings seem identical to ANE, with sudden-onset encephalopathy triggered by viral infection including influenza. The outcome, however, seems worse, with death, mild to severe developmental regression, and recurrence in half of the survivors (*33*). The simultaneous occurrence of the cases described in this study and the familial clustering may suggest either a causative role for MRV2Tou05, the patients’ genetic predisposition to such an agent, or both (*34*). Investigations are in progress to determine genetic susceptibility in the family.

The origin of several genome segments of this reassortant in swine in Asia and the relatedness of other segments to human serotype 2 reoviruses described in Asia are surprising. Possibly the MRV2Tou05 was imported by the uncle, who had just returned from Indonesia a few days before the onset of symptoms in the children and the mother. Searching for antibodies against MRV2Tou05 in serum specimens from the uncle would have been informative but no blood samples were available.

The role of reoviruses as etiologic agents for symptomatic human diseases remains controversial. They are designated as orphan viruses, and more than half of the adult population possess antibodies directed against reoviruses, which suggests that infection occurs frequently without any specific effect on human health. However, reovirus strains have been isolated from persons with serious human diseases (*35*). Indeed, an unknown reovirus strain in 1996, the T3C/96 strain in 2004, and the T2W strain in 2006 were isolated and reported from human meningitis patients, which shows that reoviruses can also cause central nervous systemic disease in humans. Melala virus, Kampar virus. and HK2369/07 virus were isolated from patients with acute respiratory infections, and BYD1, JP, and BYL strains of serotype 2 were isolated from patients with severe acute respiratory syndrome. In all these of cases, a reovirus strain with novel molecular characteristics was described, except for the 1996 case, but the characterization of the isolated strain was not reported.

## Conclusion

This study describes the entire molecular characterization of a new reovirus strain isolated from 2 familial ANE patients. Its isolation and molecular detection from patients’ samples and the specific immune response toward this type 2 strain suggest an etiologic role for this reovirus in these unexplained ANE cases. The reproduction of symptoms in an animal model and in vitro studies of the cellular interactions and apoptosis of MRV2Tou05 are needed to help clarify the exact role of this novel reovirus strain. Identifying the MRV2Tou05 reovirus sequence could contribute to the improvement of ANE diagnosis and treatment, for example, by confirming susceptibility to viral infection and clarifying the possible role of other common viruses in its pathogenicity.
